# The Use of Laser Doppler Imaging in Nitric Acid Burns: A Case Report and Literature Review

**DOI:** 10.1093/jbcr/irad044

**Published:** 2023-03-29

**Authors:** Toluwaniyin Owoso, Hadyn K N Kankam, Abdulrazak Abdulsalam, Darren Lewis

**Affiliations:** Department of Burns and Plastic Surgery, University Hospitals Birmingham NHS Foundation Trust, Birmingham, UK; Department of Burns and Plastic Surgery, University Hospitals Birmingham NHS Foundation Trust, Birmingham, UK; Institute of Inflammation and Ageing, University of Birmingham, Birmingham, UK; Department of Burns and Plastic Surgery, University Hospitals Birmingham NHS Foundation Trust, Birmingham, UK; Department of Burns and Plastic Surgery, University Hospitals Birmingham NHS Foundation Trust, Birmingham, UK

## Abstract

Laser Doppler imaging (LDI) technology has been validated to assess thermal burn depth by predicting wound healing potential. However, there is no clear evidence for its use in chemical burns. We present a case of an 8% total burn surface area (TBSA) nitric acid burn following an industrial accident, in an otherwise healthy 36-year-old man. LDI assessment was suggestive of poor healing potential of >21 days, warranting surgical management. However, conservative management was opted for based on clinical assessment as the wound eschar appeared thin and more consistent with epithelial staining. Patient follow-up confirmed a total burn healing time of two months, suggesting that the LDI assessment was accurate. A comprehensive literature review was performed using the MEDLINE (PubMed) database to identify animal or clinical studies evaluating the efficacy of LDI in chemical burns. A qualitative synthesis of our findings is presented. We identified two experimental studies in porcine models with sulfur mustard burns, each confirming the accuracy of LDI assessment when compared to the histopathology findings. Limited experimental animal studies on the use of LDI suggest similar validity in chemical burns, and this correlates with the clinical outcome in this case. However, this alone is insufficient to prove its validity and define its role in the assessment of chemical burns. Clinical trials are required to further assess and define the parameters of LDI use and efficacy in this context.

## Introduction

Chemical burns are a growing industrial and domestic concern.^1^ Nitric acid is commonly used in agricultural and metal industries.^2^ Accidental exposure has the potential to cause life-threatening injuries, including the propensity for severe chemical burns. However, nitric acid burns make up only 2% of reported chemical burns.^3^ This is reflected in the very limited medical reporting of nitric acid burns, and lack of published guidance or a widely accepted approach for the assessment and management of nitric acid burns.

Early determination of burn depth is key to estimating the wound’s healing potential and influences management choices, ultimately playing a major role in clinical, functional and aesthetic outcomes for burns patients.^4^ There is a wealth of evidence supporting the efficacy of Laser Doppler Imaging (LDI) in accurately determining burn depth in thermal burns, with clear and standardized guidance on its use in the management of thermal burns.^5^ However, there is limited evidence regarding the use of LDI in chemical burns.

We present a case report of the assessment, management and outcome of an acute nitric acid burn, and our experience of using LDI to assess burn depth in this case, supported by a literature review.

## Case Presentation

**Figure 1.**
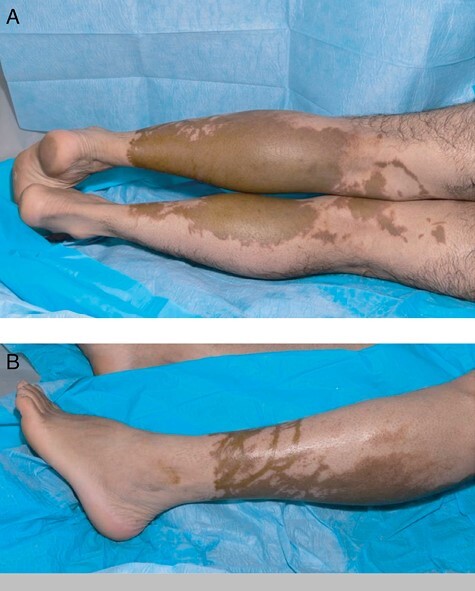
Appearance of nitric acid burns on day 2 of injury: (a) posterior aspect of bilateral legs; (b) lateral aspect of left leg.

A 36-year-old male, with no significant past medical or surgical history, sustained a bilateral leg chemical burn, secondary to an industrial accident with nitric acid. His overlying clothes (Denim jeans) were immediately removed and first aid completed with cool running water for a total of 10 minutes. An ambulance was called and he was admitted to a Regional Burns Centre in a University Teaching Hospital. Clinical examination identified an 8% total body surface area (TBSA) burn; approximately 5.5% on the posterior left leg and 2.5% on the posterior right leg, presenting as a well demarcated patch of brown discolouration to the proximal two-thirds of the posteromedial surface of both legs (Figure 1). There was no epidermolysis or exudate and the skin texture was congruent with the surrounding non-injured skin.

**Figure 2.**
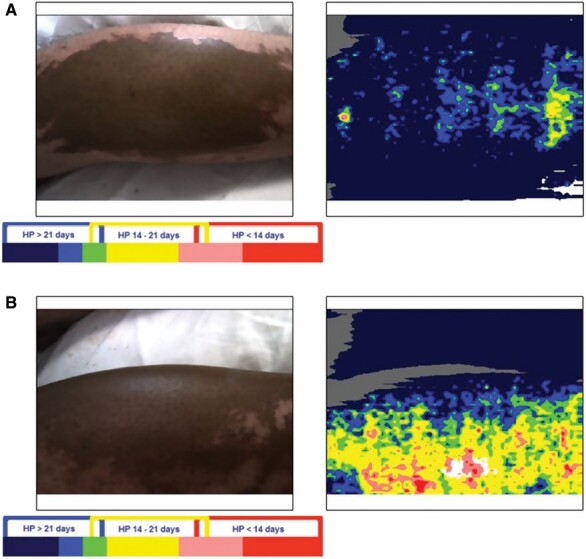
Laser Doppler images on the third day of the patient’s hospital admission: (a) posterior aspect of bilateral legs showing poor healing potential of greater than 21 days; (b) lateral aspect of left leg illustrating moderate healing potential of 14–21 days.

**Figure 3.**
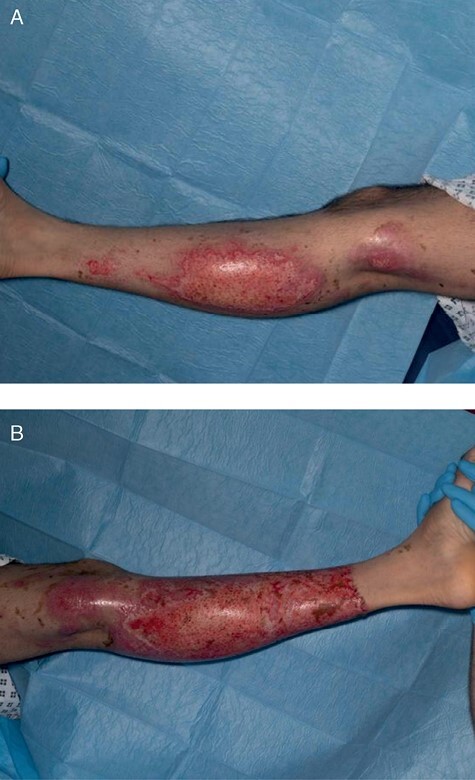
Appearance of nitric acid burns on day 17: (a) right leg; (b) left leg.

Initial management involved further irrigation with water and application of Diphoterine® spray (Prevor, Valmondois, France) two hours post-injury. Formal pain scoring was not performed but the wound pH remained at seven both pre- and post-Diphoterine® application. Diphoterine® was re-applied 48 hours following the injury due to ongoing pain. Laser doppler imaging (LDI), undertaken on the third day of admission, suggested a poor wound healing potential of more than 21 days (Figure 2a), with the exception of a small area of the wound on the lateral aspect of the left calf (Figure 2b). The LDI results did not appear to correlate with the clinical examination which suggested a more superficial injury. The thin eschar appeared as staining of the epithelium rather than a true eschar. Daily dressings of Flaminol Hydro^®^ (Flen Health, Kontich, Belgium) and Jelonet (Smith & Nephew, Tuttlingen, Germany) were applied. The patient remained systemically well, although his significant pain burden required optimization with opioid analgesia. By day 6, his wounds demonstrated some clinical signs of healing, with scattered pink islands appearing between the brown patches. By day 11, the brown patches had largely lifted to reveal underlying pink sensate re-epithelializing tissue, with a capillary refill time of less than two seconds. This was dressed with Urgotel Ag^®^ (URGO Medical, Chenove, France) and Flaminol Hydro^®^. Wound review five days later illustrated that more than 50% of the eschar had lifted and the underlying skin appearing to be mostly healed (Figure 3). Multidisciplinary team (MDT) review confirmed good healing potential and a decision was made to continue with dressings rather than surgical management. He was discharged on day 22, with the prolonged inpatient stay primarily due to issues with pain management. He did not receive any antibiotic therapy throughout his admission as there was no clinical evidence of infection and his wound microbiology was negative.

**Figure 4.**
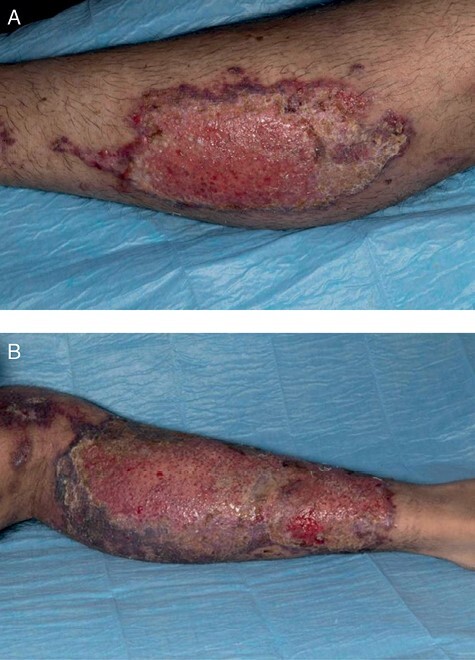
Appearance of nitric acid burns on day 30: (a) right leg; (b) left leg.

One week following discharge, the burn wound was reviewed in the outpatient nurse-led plastic surgery dressing’s clinic. On examination, the central aspect of burn wound remained unhealed, accounting for approximately 50% of the initial burn wound (Figure 4). The patient was briefly re-admitted due to ongoing issues with pain management and was subsequently discharged to the care of the community-based Burns & Plastic Surgery Outreach team for conservative wound management and regular follow-up. His most recent appointment confirmed that 90% of his wounds had healed, two months following the initial incident.

## Literature Review and Discussion

Accurate determination of burn depth directly influences therapeutic outcomes as there is significant evidence to suggest that deeper burns, which require more than 21 days to heal, have better functional and aesthetic outcomes when managed surgically with excision and soft tissue coverage.^5^ Burn depth is routinely assessed using physical parameters such as skin sensation, capillary refill and appearance, all of which rely heavily on clinical judgement.^6^ While experienced Burns and Plastic surgeons can accurately differentiate between superficial and full-thickness burns, clinical assessment of intermediate depth burns is more challenging.^7,8^

LDI uses microvascular dermal perfusion to assess burn depth and predict wound healing potential and thus is a useful adjunct to guide clinical decisions. The LDI scanner (Moor Instruments, Devon, UK) produces a red laser beam which penetrates the dermis and is reflected by moving red blood cells in the skin, causing it to undergo doppler frequency changes. The amplitude of laser doppler signal is proportional to the average speed and concentration of moving red blood cells in the tissue (flux). This flux is detected by a photo detector and processed to provide a measure of skin perfusion which is presented as colored pixels on a six-colur scale. This can be interpreted to provide three categories of healing potential: <14 days, 14-21 days, and >21 days.^9^

LDI is more than 90% accurate at predicting wound healing potential in thermal burns when used between 2 and 5 days following the initial burn injury.^9–11^ This is greater than that of clinical assessment alone, estimated to be between 64 and 76%.^7,8^ Hence, LDI is an important tool in burn care and has been recommended by the National Institute for Health and Care Excellence (NICE), for adjunctive use in clinical practice, in providing early and accurate depth assessment of thermal burns; owing to its accuracy, ease of use and non-invasive technique.^12^

**Figure 5.**
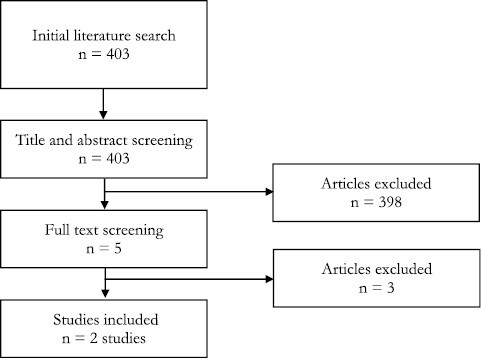
Flow diagram summary of literature search.

There is a relative scarcity of data on the use of LDI in the assessment of chemical burns. A literature search of the Medline database (Figure 5, Table 1) found only two relevant studies, both of which were experimental animal studies investigating the use of LDI in the analysis of chemical burn depth in pig models.^13,14^

**Table 1.** Summary of included studies from literature search.

Study	Year	Subject species	Sample size	Acid/alkali	Chemical	Control/comparator	Outcome measure	Outcome
Brown et al.	1998	White Pigs	8	Vesicant	Sulfur mustard or Lewisite vapor	Histopathological Analysis	Correlation with histopathology	LDI correlates with histological assessment of burn depth
Braue et al.	2007	Female Swine	6	Vesicant	Sulfur mustard	Histopathological analysis/indocyanine green fluorescence imaging (ICGF)	Correlation with histopathology	LDI and ICGF both provided good estimates of lesion depth when compared to histological analysis

Brown et al. exposed eight anesthetized pigs to a fixed concentration of Sulphur Mustard and Lewsite Mustard for six hours each. Glass fiber filter paper saturated with sulfur mustard or lewisite solution was applied to a shaved area of the dorsal skin. At specified time intervals post-exposure (2 hours, 4 hours, 6 hours, 24 hours, 3 days, and 7 days), histopathological biopsies and LDI images were obtained simultaneously. In both sulfur mustard and lewsite burns, LDI images initially demonstrated high flux at the burn wound site in the first 24 h post injury. The LDI images from day three and seven showed low flux values, corresponding to a result of low healing potential (> 21 days). These results correlated with the histopathological assessment of burn depth from the biopsies taken at these times.^13^

Similarly, Braue and colleagues exposed six anesthetized pigs to a fixed concentrations of sulfur mustard using saturated filter paper secured to various patches of shaved exposure sites on the dorsal skin, for different lengths of exposure time (2, 8, 30, and 60 minutes). At 24, 48, and 72 hours, histopathological biopsy samples were obtained and the burned sites were assessed with LDI and indocyanine green fluorescence (ICGF) imaging. Histological analysis demonstrated a positive correlation between the length of exposure to sulphur mustard and the depth of the resultant burn, which was corroborated by the findings of both LDI and ICGF imaging at 24, 48, and 72 hours post exposure. Histological samples from the 2 and 8 minutes exposure burns demonstrated evidence of a superficial and intermediate burn, respectively. LDI imaging for these areas across the three observation times showed levels of high flux, indicating a good healing potential of <21 days. Meanwhile, histological assessment of the 30 and 60 minute exposure burns demonstrated a deep dermal and full thickness burn, respectively. At all observation times, LDI images for both the 30 and 60 minute exposure burns showed low flux, indicative of poor healing potential >21 days.^14^

In both experimental studies, the LDI findings corresponded with histological findings, which is an accurate measure of burn depth. This supports the validity of LDI in determination of burn depth in chemical burns. This is in accordance with the experiential findings from this case report. In this case, LDI suggested poor healing potential of >21 days but clinical assessment was in favor of a good healing potential; therefore conservative management was implemented. However, the burn wounds remained 50% unhealed at one month post-injury.

In this case, clinical judgment was affected by the unusual presentation of the burn. The brown patch appearance, which is unique to nitric acid burns,^3^ made it difficult to assess burn depth clinically. It was felt that the relatively thin eschar, initially thought to be stained epithelium, was affecting the results. A significant proportion of the underlying wound bed was only visible on the eleventh day after the injury. By day 17, the underlying wound bed appeared to be islands of re-epithelializing tissue, which re-enforced the decision to continue with conservative management. However, contrary to clinical expectation, the healing process was longer than anticipated. The disparity between clinical assessment and clinical outcome could have been due to various external factors including individual genetics and general healing potential or poor compliance with dressings. This could also have been the result of intrinsic pathophysiology of nitric acid burns; however, further work is necessary to provide more insight into this mechanistic process.

While the results of this case and the two experimental studies support a potential role of LDI use in chemical burns, these alone are not sufficient to validate routine clinical use in this setting. The presence of only two relevant studies in the literature, both with small sample sizes, demonstrates the limited evidence in the literature. Moreover, questions regarding the ecological validity of these experiments provides uncertainties about whether the results can be reliably extrapolated to human cohorts in the clinical setting. In both studies, chemical exposure was clinically controlled by exposing a small, precise section of shaved and cleaned skin to the vesicant chemicals. It is difficult to know if this level of controlled exposure in pig models can accurately mimic the accidental or deliberate exposure to chemicals that we observe in clinical practice. In addition, both studies were using vesicant chemicals as the inoculating chemical. Vesicant chemicals are an area of interest in research as they have been historically used as chemical warfare, usually in vapor form, during World War 1.^15^ Exposure to such chemicals is highly unlikely in western domestic or industrial settings. Hence, it is difficult to know if the pathophysiology of injury and healing are the same in vesicant burns compared to the usual chemical burns generally encountered in civilian populations.

Further studies are required to confirm the validity of LDI use in chemical burns. This should be clinical and prospective in nature, assessing LDI in the context of a variety of chemical agents in order to verify the robustness of its capabilities. Additionally, there must be clarification regarding the extent of its use and limitations in this context, with regards to various factors such as: technique for LDI use, time frame of use and interpretation of results. This is as all of the current practice regarding LDI is based on thermal burns and may not be optimized for assessing chemical burns.

## Conclusions

Early and accurate burn depth assessment is key for deciding on appropriate clinical management and significantly influences therapeutic outcomes. The use of LDI is clinically validated and endorsed by NICE guidance in thermal burns, between two and five days post-injury, to accurately assess burn depth and healing potential. Limited experimental animal studies suggest similar validity in chemical burns, and this correlates with the clinical outcome in this case. However this alone is insufficient to prove the validity of LDI and define its role in the assessment of chemical burns. Clinical trials are needed to assess the validity of LDI in chemical burns and define the parameters of its use and efficacy in this context.

## Notes

Funding: Hadyn K.N. Kankam is a National Institute for Health Research (NIHR) Academic Clinical Fellow. No direct funding was received for this article. The views expressed are those of the authors and not necessarily those of the NIHR or the Department of Health and Social Care.

## References

[1] Walsh K , Hughes I, Dheansa B. Management of chemical burns. Br J Hosp Med 2022;83:1–12. 10.12968/hmed.2020.0056 35377199

[2] Public Health England. PHE Centre for Radiation, Chemical and Environmental Hazards. Nitric Acid: Toxicology Overview. 2018. Last accessed on 31 March 2023: https://assets.publishing.service.gov.uk/government/uploads/system/uploads/attachment_data/file/673594/Nitric_acid_TO_120118.pdf

[3] Kolios L , Striepling E, Kolios G et al. The nitric acid burn trauma of the skin. Journal of Plastic, Reconstructive & Aesthetic Surgery 2010;63:e358–63. 10.1016/j.bjps.2009.09.001 19875347

[4] Monstrey S , Hoeksema H, Verbelen J, Pirayesh A, Blondeel P. Assessment of burn depth and burn wound healing potential. Burns 2008;34:761–9. 18511202 10.1016/j.burns.2008.01.009

[5] Zuo KJ , Medina A, Tredget EE. Important developments in burn care. Plast Reconstr Surg 2017;139:120e–38e. 10.1097/PRS.0000000000002908 28027250

[6] Heimbach D , Engrav L, Grube B, Marvin J. Burn depth: a review. World J Surg 1992;16:10–5. 1290249 10.1007/BF02067108

[7] Jaskille AD , Shupp JW, Jordan MH, Jeng James C. Critical review of burn depth assessment techniques: Part I. Historical review. J Burn Care Res 2009;30:937–47. 19898102 10.1097/BCR.0b013e3181c07f21

[8] Heimbach DM , Afromowitz MA, Engrav LH, Marvin JA, Perry B. Burn depth estimation - man or machine. J Trauma 1984;24:373–8. 6371255

[9] Hoeksema H , Van de Sijpe K, Tondu T et al. Accuracy of early burn depth assessment by laser Doppler imaging on different days post burn. Burns 2009;35:36–45. 18952377 10.1016/j.burns.2008.08.011

[10] Hoeksema H , Baker RD, Holland AJA et al. A new, fast LDI for assessment of burns: a multi-centre clinical evaluation. Burns 2014;40:1274–82. 24996246 10.1016/j.burns.2014.04.024

[11] Stewart TL , Ball B, Schembri PJ et al.; Wound Healing Research Group. The use of laser Doppler imaging as a predictor of burn depth and hypertrophic scar postburn injury. J Burn Care Res 2012 Dec;33:764–71. 22955162 10.1097/BCR.0b013e318257db36

[12] NICE Guideline. moorLDLS-BI for burn depth assessment. 2021. Last accessed on 31 March 2023: https://www.nice.org.uk/advice/mib251/resources/moorldlsbi-for-burn-depth-assessment-pdf-2285965688374213

[13] Brown RF , Rice P, Bennett NJ. The use of laser Doppler imaging as an aid in clinical management decision making in the treatment of vesicant burns. Burns 1998;24:692–8. 9915668 10.1016/s0305-4179(98)00105-3

[14] Braue EH , Graham JS, Doxzon BF et al. Noninvasive methods for determining lesion depth from vesicant exposure. J Burn Care Res 2007;28:275–85. 17351445 10.1097/BCR.0B013E318031A1A8

[15] Mellor SG , Rice P, Cooper GJ. Vesicant burns. Br J Plast Surg 1991;44:434–7. 1933115 10.1016/0007-1226(91)90202-u

